# Supplementary material for: Identification of Small Molecule Ligand Binding Sites On and In the ARNT PAS-B Domain
Source: bioRxiv. 2023 Nov 5:2023.11.03.565595. Preprint. [Version 1] doi: 10.1101/2023.11.03.565595 (PMC10635134; doi:10.1101/2023.11.03.565595)
Supplement: Supplement 1 [file media-1.pdf]

Supporting Information

Identification of Small Molecule Ligand Binding Sites On and In the ARNT PAS-B Domain

Xingjian Xu, Leandro Pimentel Marcelino, Denize C. Favaro, Marion L. Silvestrini, Riccardo Solazzo, Lillian T. Chong, Kevin H. Gardner

Contents:

Tables S1-S4
Figures S1-S9

Table S1 NOE Assignments for ARNT PAS-B/KG-655

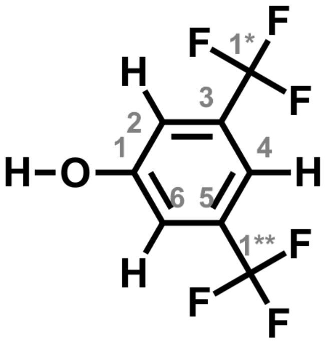

3,5-Bis(trifluoromethyl)phenol

Protein Assignment	Ligand Assignment	Distance Range (Å)
I396HD11	H4	1.8-5.0
I396HD11	H2/H6	1.8-5.0
I396HG21	H2/H6	1.8-2.8
# I458HG21	H4	1.8-2.8
I457HG21	H4	1.8-3.5
V425HG11	H2/H6	1.8-3.5
* V381HG11	H4	1.8-3.5
V415HG11	H4	1.8-3.5
L418HD21	H4	1.8-5.0
L418HD21	H2/H6	1.8-3.5
L408HD11	H2/H6	1.8-2.8
V415HG21	H4	1.8-5.0
L423HD11	H2/H6	1.8-3.5
L418HG	H2/H6	1.8-3.5

Surface-facing residues marked with #. Outliers (do not belong to either binding site) are marked with *.

Table S2 NOE Assignments for ARNT PAS-B/KG-279

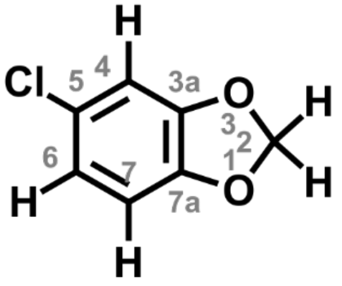

5-chloro-1,3-benzodioxole

Protein Assignment	Ligand Assignment	Distance Range (Å)
I396HD11	H4	1.8-5.0
I396HD11	H6	1.8-3.5
I396HD11	H7	1.8-3.5
I457HD11	H4	1.8-5.0
I457HD11	H6	1.8-5.0
I457HD11	H7	1.8-5.0
I457HD11	H2a/H2b	1.8-2.8
I396HG21	H4	1.8-3.5
I396HG21	H6	1.8-3.5
I396HG21	H7	1.8-2.8
# I458HG21	H4	1.8-5.0
# I458HG21	H6	1.8-5.0
# I458HG21	H7	1.8-5.0
I457HG21	H4	1.8-5.0
I457HG21	H6	1.8-5.0
I457HG21	H7	1.8-5.0
I457HG21	H2a/H2b	1.8-5.0
V425HG21	H4	1.8-2.8
V425HG21	H6	1.8-3.5
V425HG21	H7	1.8-5.0
V425HG21	H2a/H2b	1.8-5.0
L418HD21	H4	1.8-3.5
L418HD21	H6	1.8-2.8
L418HD21	H7	1.8-3.5
L408HD11	H4	1.8-3.5
L408HD11	H6	1.8-3.5
L408HD11	H7	1.8-5.0
V415HG21	H4	1.8-3.5
L408HD21	H4	1.8-5.0
L408HD21	H6	1.8-5.0
L408HG	H4	1.8-3.5

Surface-facing residues marked with #.

Table S3 GAFF2 atomic charges derived for KG-655 drug ligand. Atom names in the structure of the ligand correspond to those used for ligand parameterization. Note that these atom names differ from labels by IPUAC locants in Tables S1 and S2.

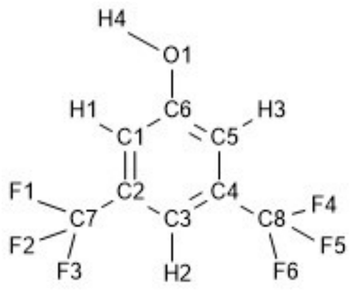

Atom Name	Atom Type	Partial Atomic Charges
C1/C5	CA	-0.344626
H1/H3	HA	0.208731
C2/C4	CA	-0.020023
C7/C8	C3	0.660231
F1/F2/F3	F	-0.214117
C3	CA	-0.193060
H2	HA	0.167975
F4/F5/F6	F	-0.214117
C6	CA	0.458901
O1	OH	-0.549457
H4	HO	0.391717

Table S4 GAFF2 atomic charges derived for KG-279 drug ligand. Atom names in the structure of the ligand correspond to those used for ligand parameterization. Note that these atom names differ from labels by IPUAC locants in Tables S1 and S2.

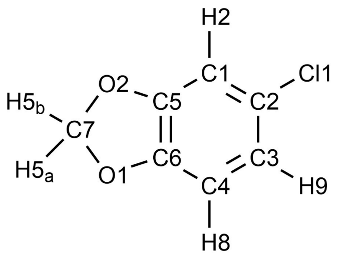

Atom Name	Atom Type	Partial Atomic Charges
C1	CA	-0.238490
H2	HA	0.203597
C5	CA	0.287911
C2	CA	-0.067733
Cl1	CL	-0.101533
C3	CA	-0.042623
H9	HA	0.150656
C4	CA	-0.431844
H8	HA	0.239701
C6	CA	0.384687
O1	OS	-0.447371
C7	C3	0.388408
H5a/H5b	H2	0.055106
O2	OS	-0.435578

SI Figures

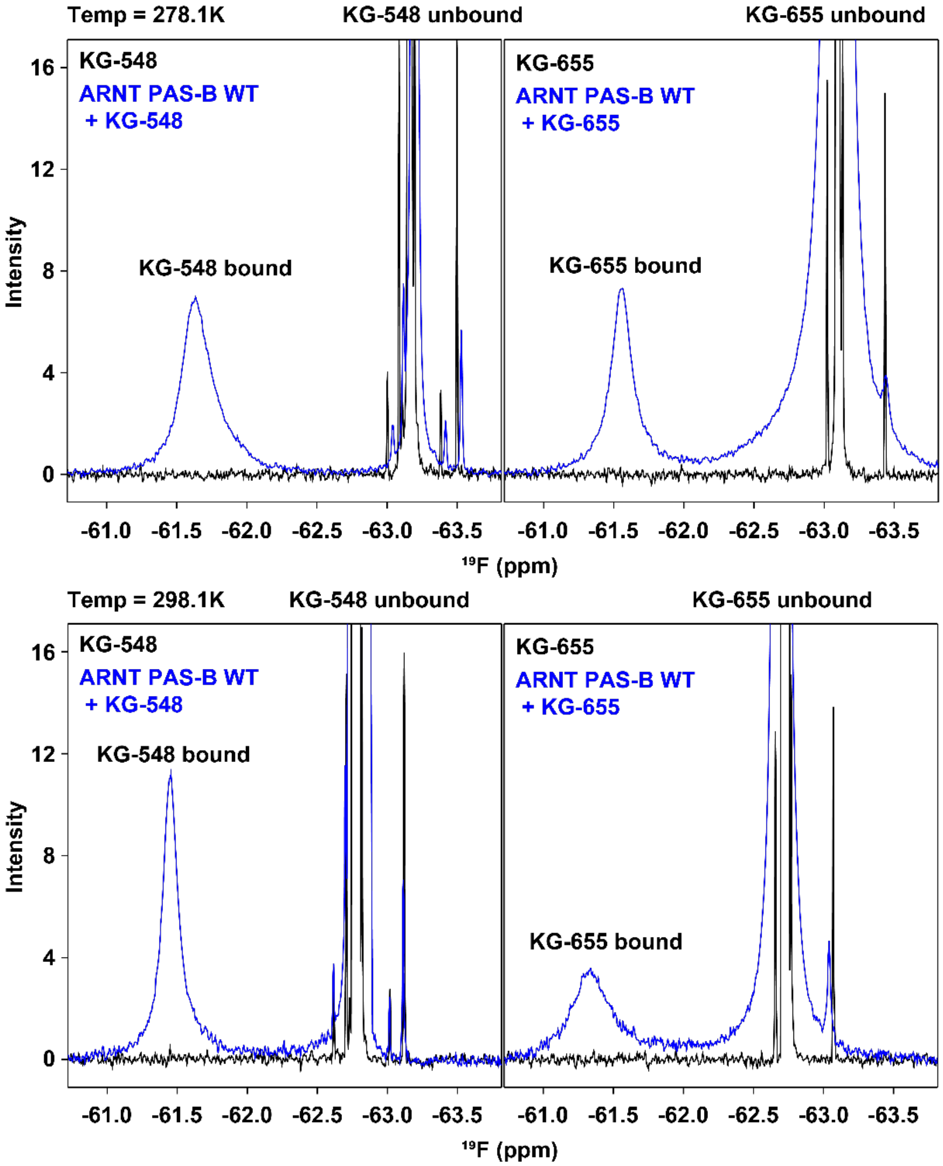

Figure S1. Evaluation of KG-548 and KG-655 ligand interactions with ARNT PAS-B using 1D ^{19}F NMR at 278K.1 and 298.1K.

The surface-bound spectra of both ligands showed an additional peak between -61 and -61.5 ppm. The free ligand peak of KG-655 was also broadened in the presence of ARNT PAS-B, indicating a possible second binding mode.

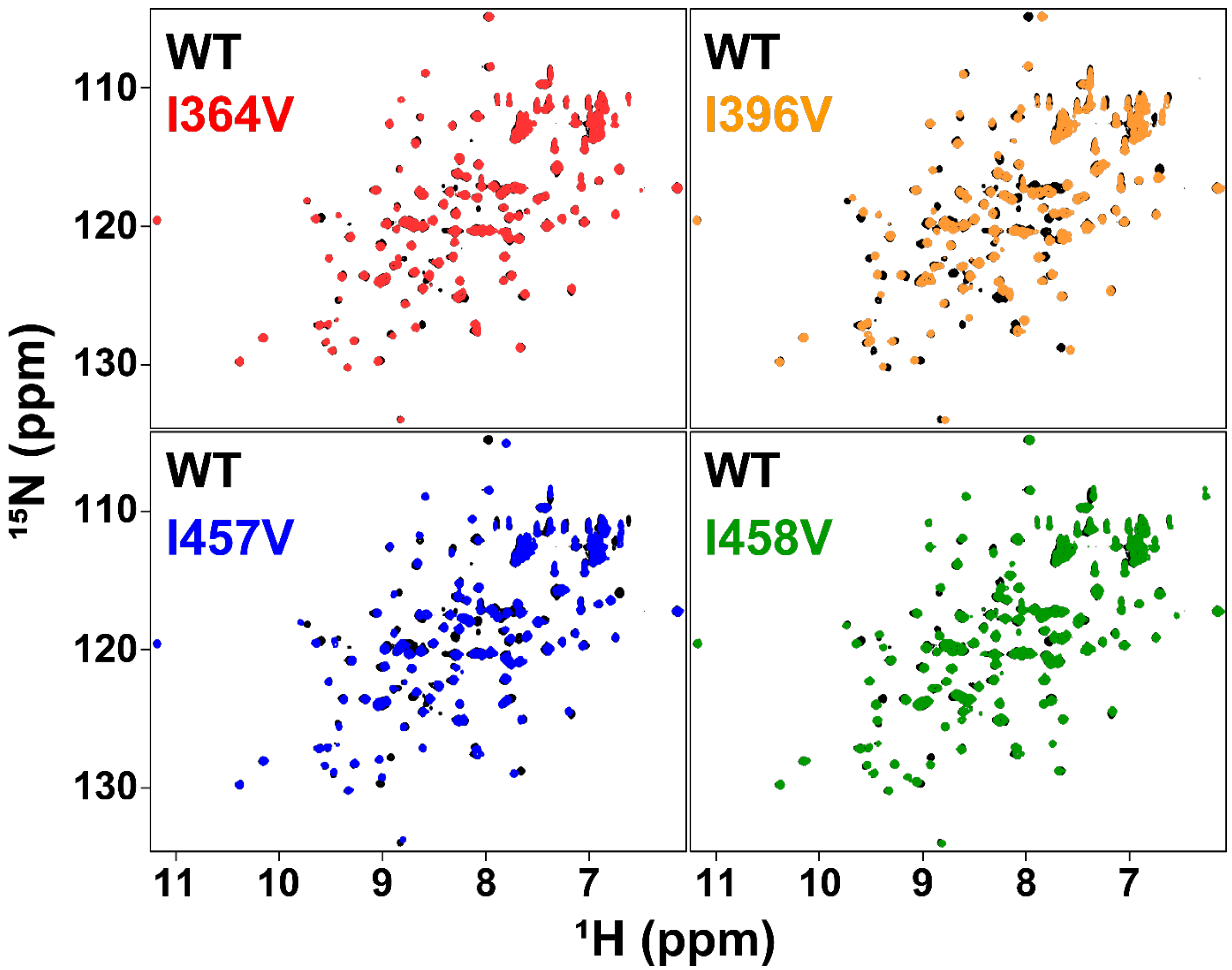

Figure S2. Comparisons of the four Ile to Val mutants to the WT ARNT PAS-B protein. $^{15}N/^{1}H$-HSQC spectra of the four Ile to Val ARNT PAS-B mutants (250 μM) indicated these mutants were folded and adopted similar structures to the WT protein, as evident by good chemical shift dispersion and overlap with the spectrum of the WT ARNT PAS-B.

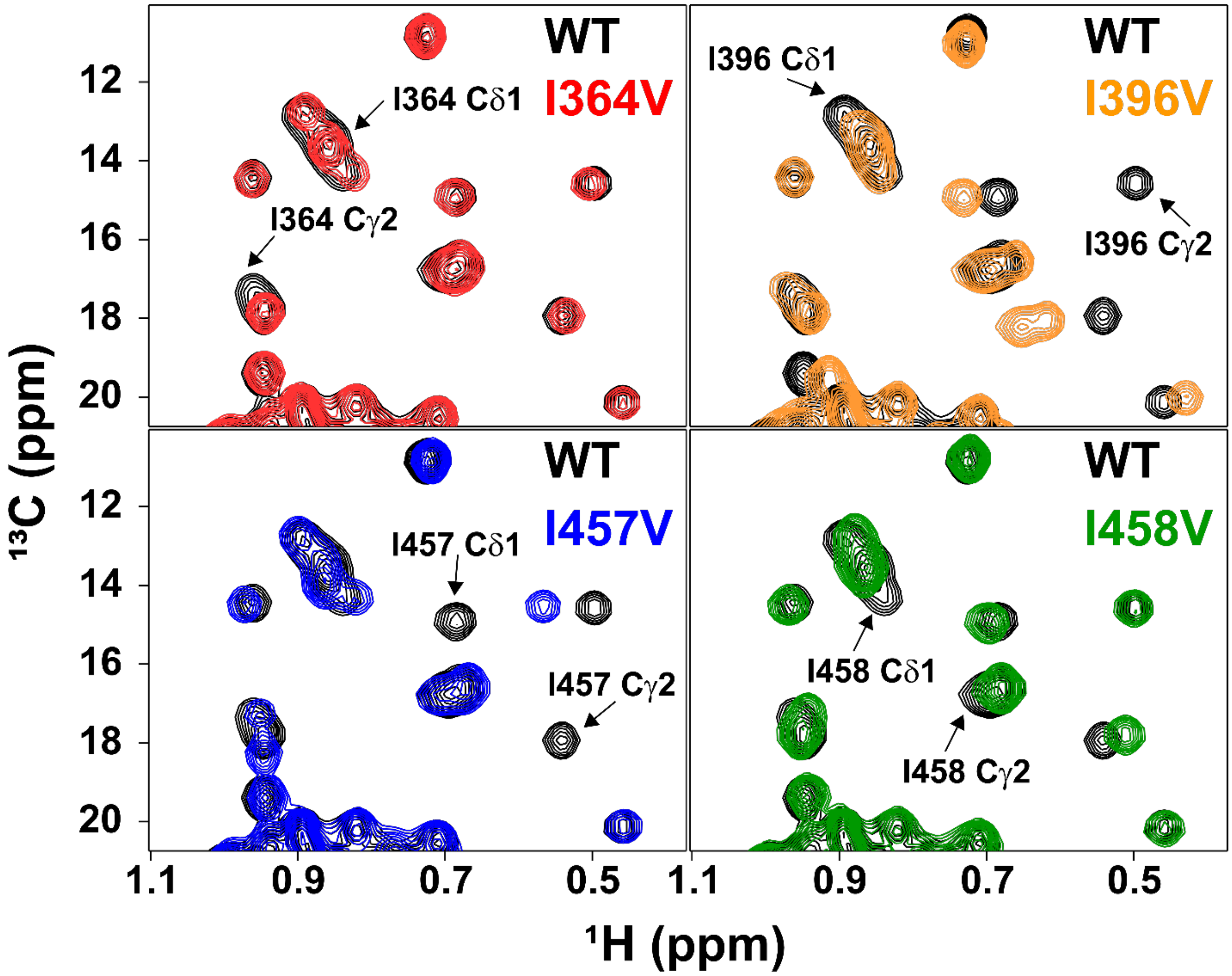

Figure S3. $^{13}C/^{1}H$-HSQC spectra of the ARNT PAS-B mutants compared with the wildtype (WT) protein.

$^{13}C/^{1}H$-HSQC spectra of the four Ile to Val ARNT PAS-B mutants (250 μM) were used to confirm the Ile methyl assignments. The methyl cross-peak locations of the mutated residues are marked with arrows. Peaks other than the mutated Ile residues showed good overlap with the spectrum of the WT protein.

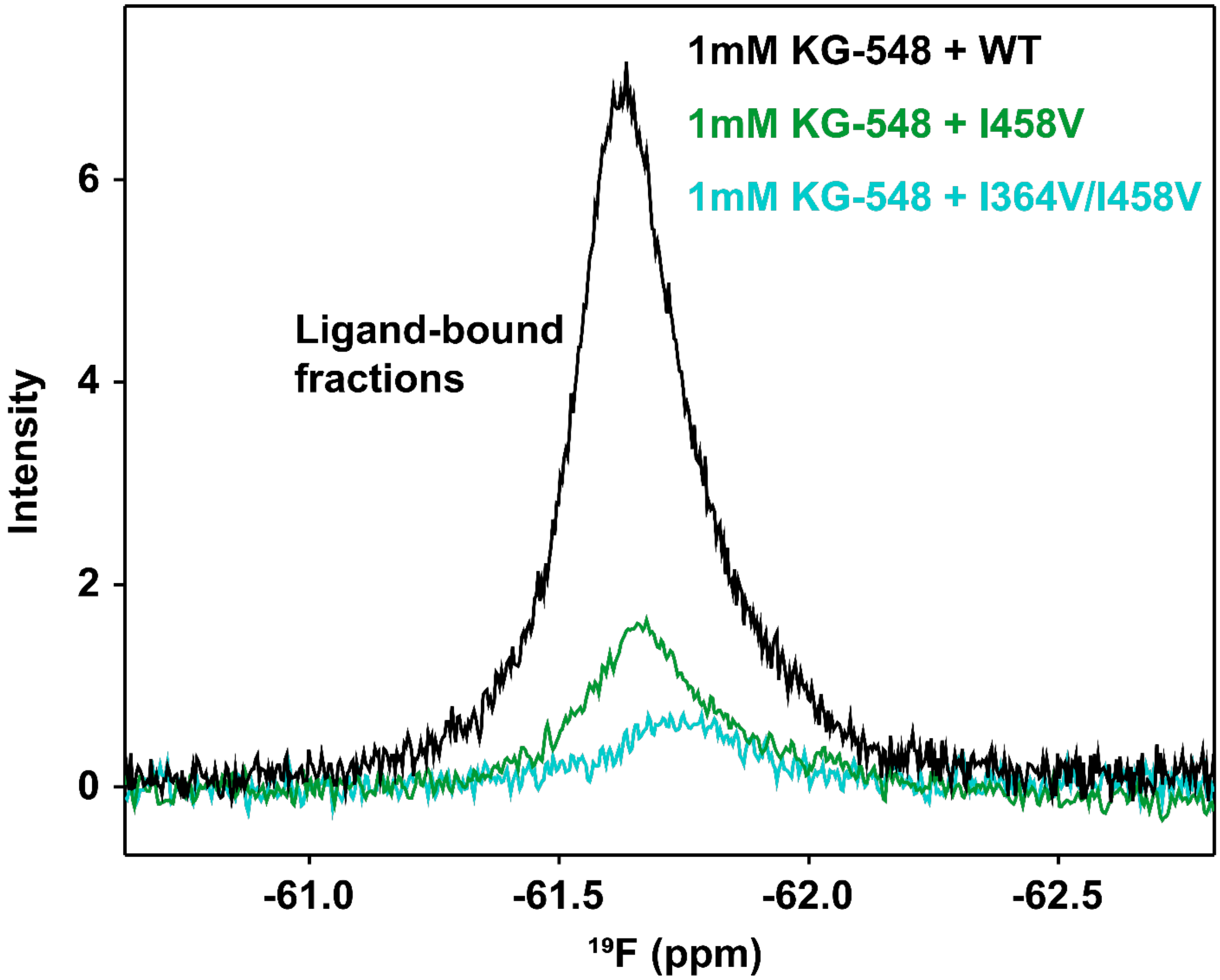

Figure S4. I364V/I458V double mutation further disrupts surface binding.
^{19}F-spectra of 1 mM KG-548 mixed with 250 μM ARNT PAS-B WT, I458V, or I364V/I458V double mutant, zoomed in on the bound-fraction of the ligand. Mutation I458V substantially decreased binding affinity, with the I364V/I458V double mutation resulting in the lowest amount of bound ligand.

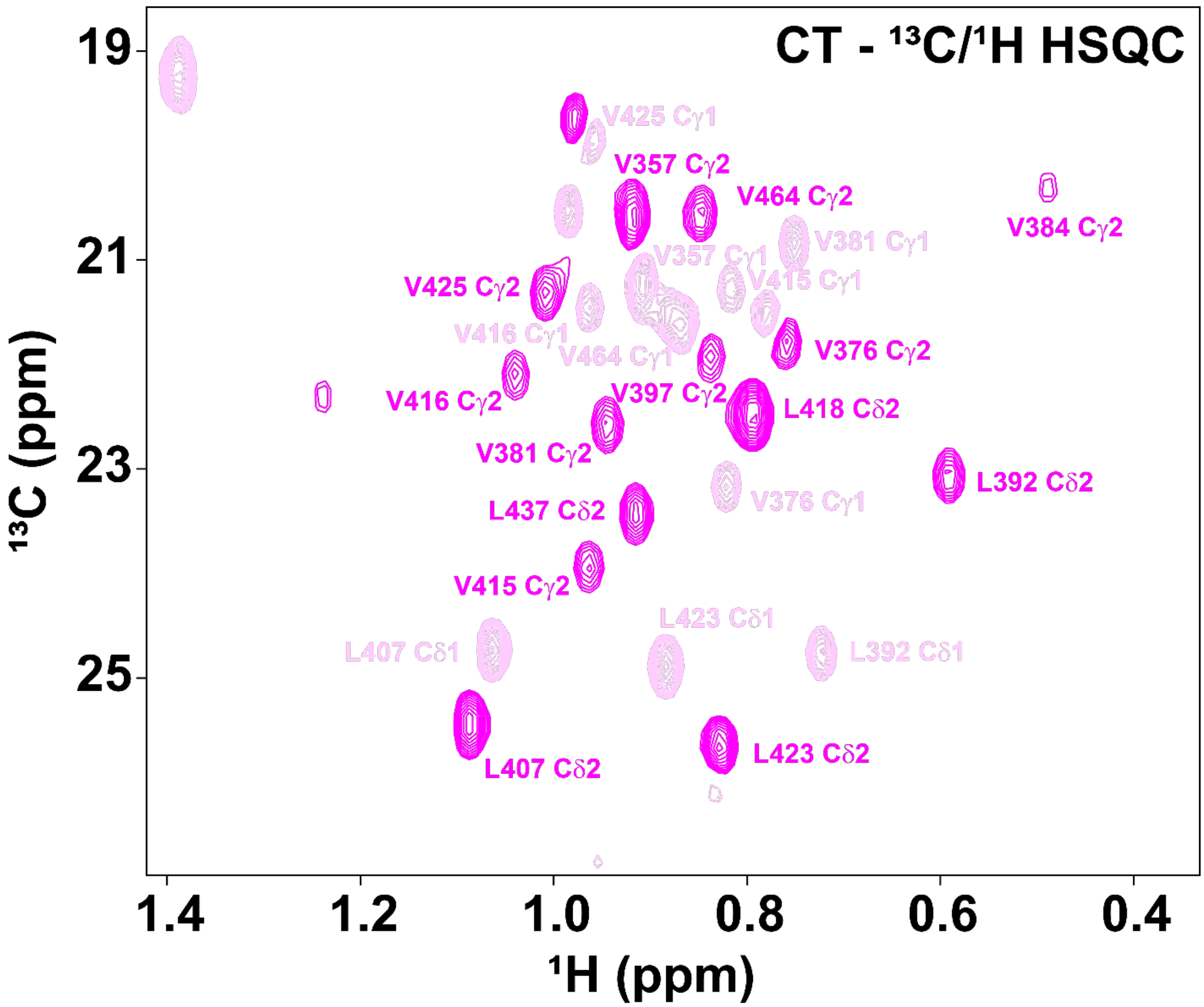

Figure S5. Stereospecific assignments of Leu and Val methyl resonances.
Pro-R methyl groups (γ1 and δ1 for Val and Leu, respectively) yield negative signals in this constant time (CT) -^{13}C/^{1}H HSQC experiment. Pro-S methyl groups (γ2 and δ2 for Val and Leu, respectively) yield positive signals. Pro-S methyl groups are the migrating methyl groups. When they are ^{13}C labeled, the adjoining carbons are highly unlikely also to be labeled. Signs of the signals are determined by checking the signs of the Met methyl peaks, which are positives here.

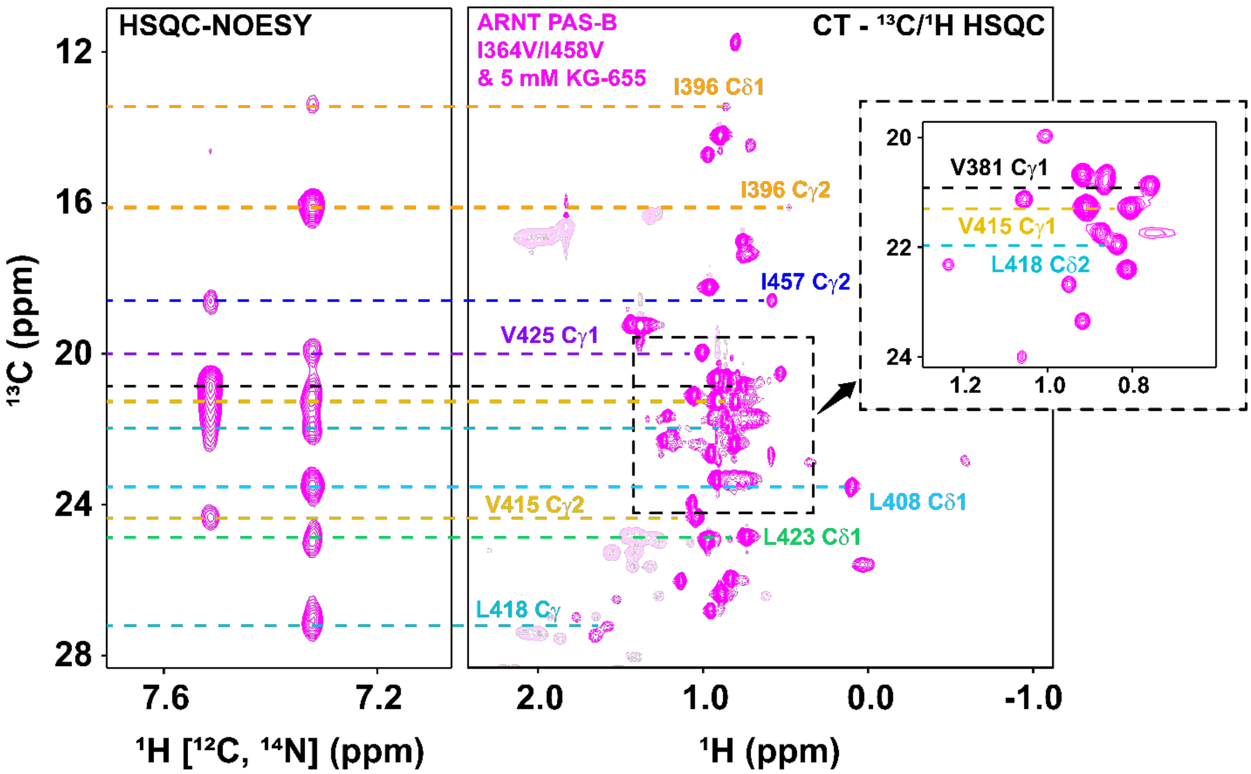

Figure S6. The surface and internal binding modes of KG-655 are independent of each other.

A double-filtered HSQC-NOESY experiment was performed using KG-655 and ARNT PAS-B I364V/I458V double mutant. Heteronuclear NOE correlations between KG-655 and methyl groups of all of the internal-facing protein residues identified previously using the WT ARNT PAS-B remain unaffected.

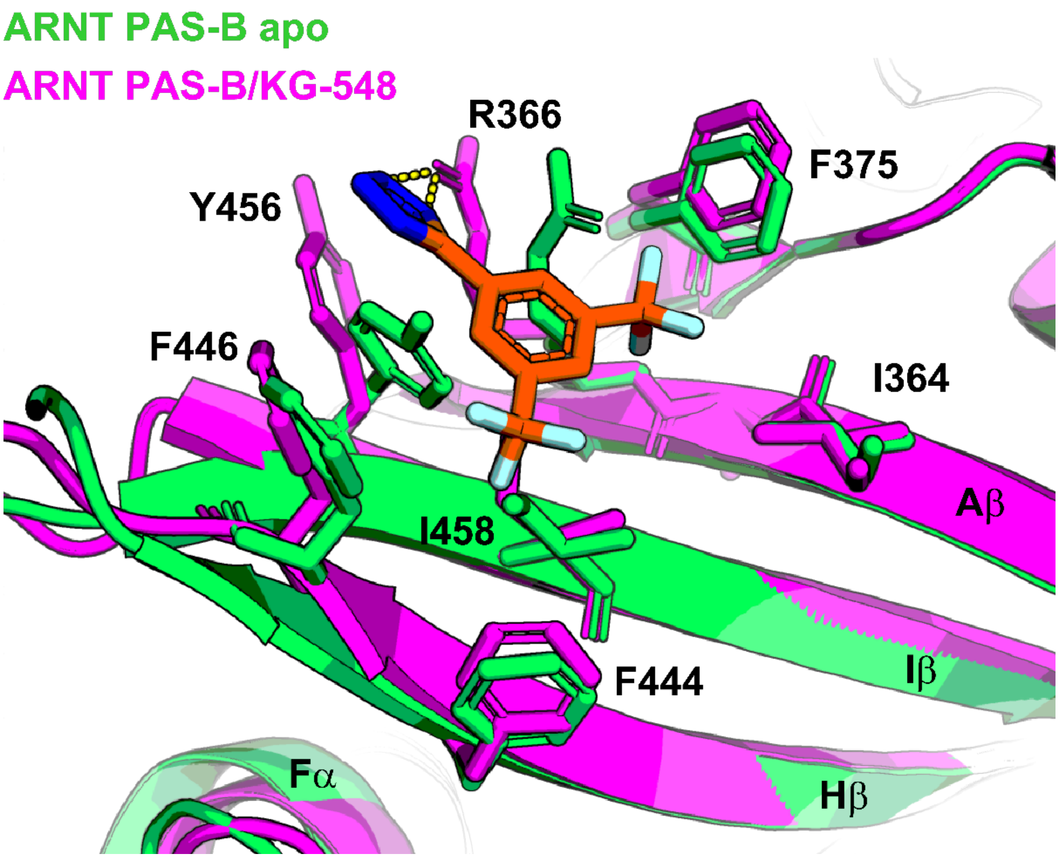

Figure S7. Comparison between ARNT PAS-B apo and KG-548 bound states.
Superimposition of the crystal structure of the ARNT PAS-B apo (PDB: 4EQ1, lime green) and KG-548 bound state (PDB: 8G4A, magenta), with residues within 5Å of the ligand shown in sticks. Residue Y456 moves away from KG-548 in the ligand-bound state to prevent clashes. R366 also reorients and forms polar contacts with the tetrazole group of KG-548 (yellow dashed lines).

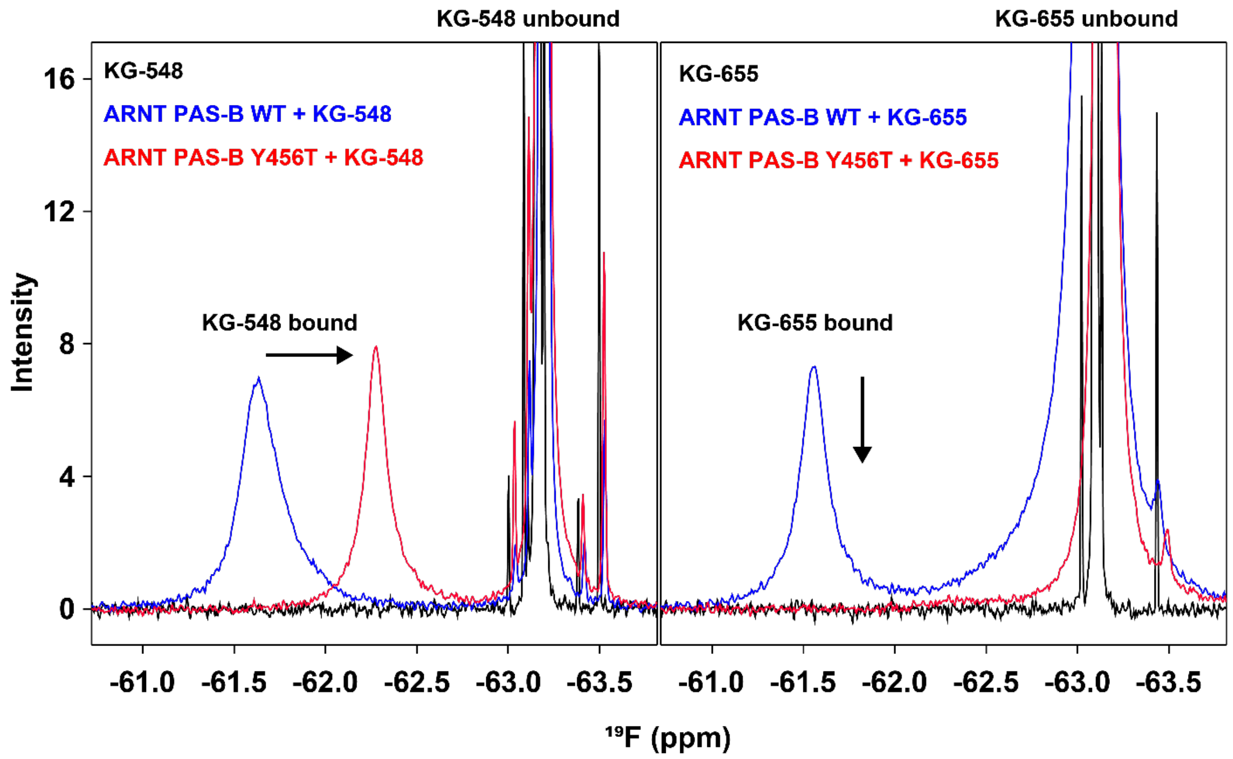

Figure S8. Mutation Y456T disrupts the surface binding of KG-655.
^{19}F spectra of ligands KG-548 or KG-655 (1 mM) mixed with ARNT PAS-B WT and Y456T (250 µM), collected at 278.1K. Mutation Y456T affected the surface binding of the two ligands differently, shifting the ligand-bound peak of KG-548 upfield by 0.65 ppm while completely abolishing the surface binding of KG-655. The internal binding mode of KG-655 also appeared to be affected (less broadening of the peak at -63.1 ppm) by the mutation.

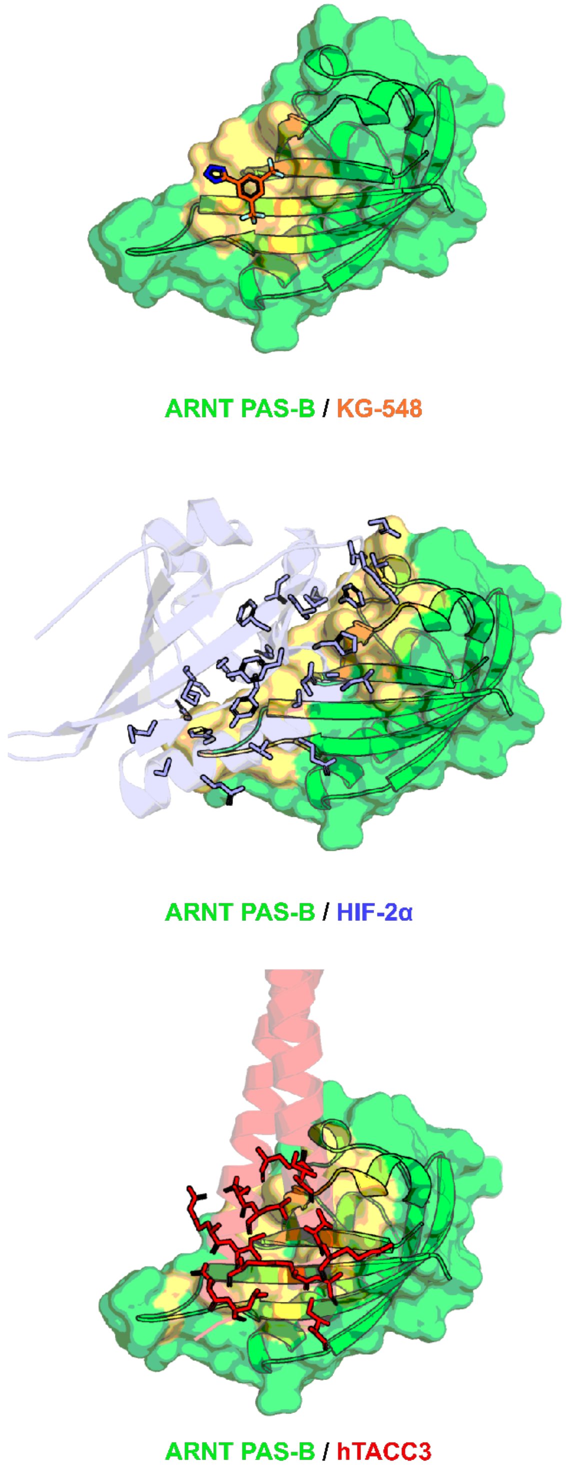

Figure S9. The ARNT PAS-B/KG-548 interface is an important surface binding 'hotspot'. The same β-sheet surface has also been shown to interact with HIF-2α PAS-B and TACC3 coactivator.
